# Cabbage Exosome-Like Nanoparticles Encapsulating Small Noncoding tsRNA Prevent Postinjury Arterial Restenosis

**DOI:** 10.34133/research.1019

**Published:** 2025-11-26

**Authors:** Xiangyu Liu, Xinxing Wang, Jian Li, Zhongjie Zhao, Yan Liu, Xiaolu Li, Wentao Wang, Qianqian Wang, Xiaozhi Sun, Mingjin Guo, Tao Yu, Yongxin Li

**Affiliations:** ^1^ Department of Vascular Surgery, The Affiliated Hospital of Qingdao University, Qingdao 266000, Shandong, People’s Republic of China.; ^2^ Department of Cardiology, The Affiliated Hospital of Qingdao University, Qingdao 266000, Shandong, People’s Republic of China.; ^3^ Institute for Translational Medicine, The Affiliated Hospital of Qingdao University, Qingdao 266021, Shandong, People’s Republic of China.

## Abstract

Postinjury restenosis is a common complication of peripheral arterial disease treated via endovascular techniques. Its pathogenesis mainly involves neointimal hyperplasia and persistent inflammation. Although antiproliferative drugs used clinically can temporarily slow restenosis, their effects are limited by short action duration and lack of precise regulation. In this investigation, tRF-49:69-chrM.Trp-TCA (tRF-Trp-TCA) was identified through sequencing data from an animal restenosis model, and its regulatory effects on endothelial cell migration and inflammation were confirmed. Additionally, we discovered that cabbage exosome-like nanoparticles (CELNs) could precisely target injured blood vessels in vivo, enhance the stability of nucleic acid therapeutics, and more effectively inhibit neointimal hyperplasia in a carotid artery balloon injury model. Our results demonstrated that tRF-Trp-TCA is crucial in restenosis induced by arterial injury and CELNs loaded with tRF-Trp-TCA effectively inhibit neointimal hyperplasia following carotid artery injury in rats, showing good biocompatibility. This study has, for the first time, identified the target tRF-Trp-TCA for treating restenosis after vascular injury and has also, for the first time, used CELNs as the delivery system. This discovery could provide new insights for noninvasive treatments or mitigation of restenosis post-endovascular therapy.

## Introduction

Percutaneous transluminal angioplasty effectively revascularizes the superficial femoral artery, achieving initial technical success rates above 95% with minimal complications [1–3]. In-stent restenosis (ISR) remains a critical limitation affecting the durability of stent-based interventions for lower limb ischemia, as ISR occurs in 40% to 60% of cases within 1 year [4,5]. Researchers indicate that during endovascular therapy, the application of scaffolds and balloons exerts pressure on plaques, resulting in damage to the intimal and medial layers of the vascular wall. This damage instigates an acute inflammatory response, which releases tissue factors that provoke localized inflammation and the clustering of inflammatory cells, ultimately leading to luminal stenosis [6]. Upon stimulation with tumor necrosis factor-α (TNF-α), endothelial cells increase the expression of adhesion molecules, including vascular cell adhesion molecule-1 (VCAM-1) and intercellular adhesion molecule-1 (ICAM-1). An alternative hypothesis for ISR attributes the condition to delayed endothelial repair. Drug-eluting stents hinder the repair of the damaged intima, resulting in inadequate endothelial coverage [7]. Prolonged inhibition of endothelial repair disrupts endothelial homeostasis, increasing susceptibility to white blood cell and platelet adhesion, and promoting lipid accumulation on the vessel walls. Once the drug is fully released from the stent, the absence of endogenous growth suppression within vessels, coupled with stent-triggered mitogenic activation, can drive pathological hyperplasia of endothelial and smooth muscle cells—a central mechanism underlying ISR development. Additionally, studies have shown that irregular stent protrusion and rupture post-stenting are linked to neointimal formation [8,9]. Therefore, there is an urgent need for the development of more noninvasive and targeted therapeutic agents.

Recent advancements in deep sequencing technology have led to the discovery of a novel class of small noncoding RNAs known as transfer RNA-derived small RNAs (tsRNAs) in a variety of organisms [10,11]. Transfer RNAs (tRNAs) primarily function in protein biosynthesis by delivering amino acids to the ribosome [12]. Cleavage within the tRNA anticodon loop produces tRNA-derived stress-induced RNAs (tiRNAs), whereas shorter fragments are termed tRNA-derived fragments (tRFs) [13,14]. Both tiRNAs and tRFs are essential for various biological regulatory processes and are implicated in several diseases. Emerging evidence shows that environmental stressors—such as starvation, oxidative stress, and hypoxia—trigger site-specific cleavage of endogenous tRNAs to generate small fragments [15–17]. Notably, tiRNAs and tRFs are associated with neurodegenerative diseases, metabolic disorders, stress-induced damage, and cancer [18–21]. Their mechanisms include gene silencing, modulation of protein translation, and competitive binding to critical proteins [22,23]. Furthermore, accumulating studies indicate that tsRNAs contribute to cardiovascular disease (CVD) by regulating vascular smooth muscle cell (VSMC) proliferation [24,25]. Increased levels of tRF-Glu-CTC promote the proliferation and migration of VSMCs, while reducing its expression following balloon injury in the rat carotid artery leads to a decrease in the neointimal area [26]. tRF-Gly-CCC is crucial for sustaining VSMC functionality and modulating inflammatory cell responses, and reducing the mortality of aortic dissection/aneurysm [27].

Plant-derived exosome-like nanoparticles (PELNs) are valuable natural compounds obtained from the multivesicular bodies of various edible plants, such as fruits and vegetables [28]. These nanoparticles share morphological traits, secretion mechanisms, and content compositions with mammalian extracellular vesicles [29–31]. Conversely, PELNs offer the benefits of high yield and cost-effectiveness. Plants secrete extracellular components, including miRNA, bioactive lipids, mRNA, and proteins as PELNs [32]. PELNs act as extracellular messengers, facilitating intercellular communication and offering biodefense against diseases. They also possess antioxidant, anti-inflammatory, and regenerative properties [33,34]. Recent investigations have focused extensively on PELNs for drug delivery applications, owing to their safety profile, biocompatibility, biodegradability, and negligible effects on intestinal barrier integrity and organ toxicity [35–38]. Broccoli, pomegranate, apple, and orange ELNs with miRNAs like miR159, miR162, miR166, and miR396 can serve as nanocarriers for functional miRNAs applicable in RNA-based therapies [39]. Oral delivery of green tea-derived ELNs encapsulating nucleic acid therapeutics enables targeted treatment for aortic dissection [40]. Their diverse sources make PELNs suitable for mass production. Advances in science and technology are enhancing extraction and separation techniques for PELNs, enabling the development of nanoplatforms for bioactive substance delivery and anti-inflammatory therapies, addressing modern clinical challenges [41–43].

In this study, we first identified the key regulatory tRF-49:69-chrM.Trp-TCA (tRF-Trp-TCA) in animal models using transcriptome analysis, which is validated to be intricately linked to endothelial cell inflammation, migration, and neointimal hyperplasia in restenosis models. Simultaneously, we isolated and purified cabbage exosome-like nanoparticles (CELNs), which possess intrinsic anti-inflammatory properties, to serve as carriers for the protection and delivery of nucleic acid therapeutics [44]. We then encapsulated the tsRNA within CELNs. The innate inflammatory chemotaxis of CELNs ensures precise delivery of nucleic acid therapeutics to the lesion site, facilitating disease remission. Our extensive evaluation of CELN^tsRNA^ targeting, therapeutic efficacy, and biosafety demonstrated its potential for targeted vascular injury treatment, offering a novel strategy for developing innovative vascular therapeutics.

## Results

### tRF-Trp-TCA screening and functional verification

**Fig. 1.**
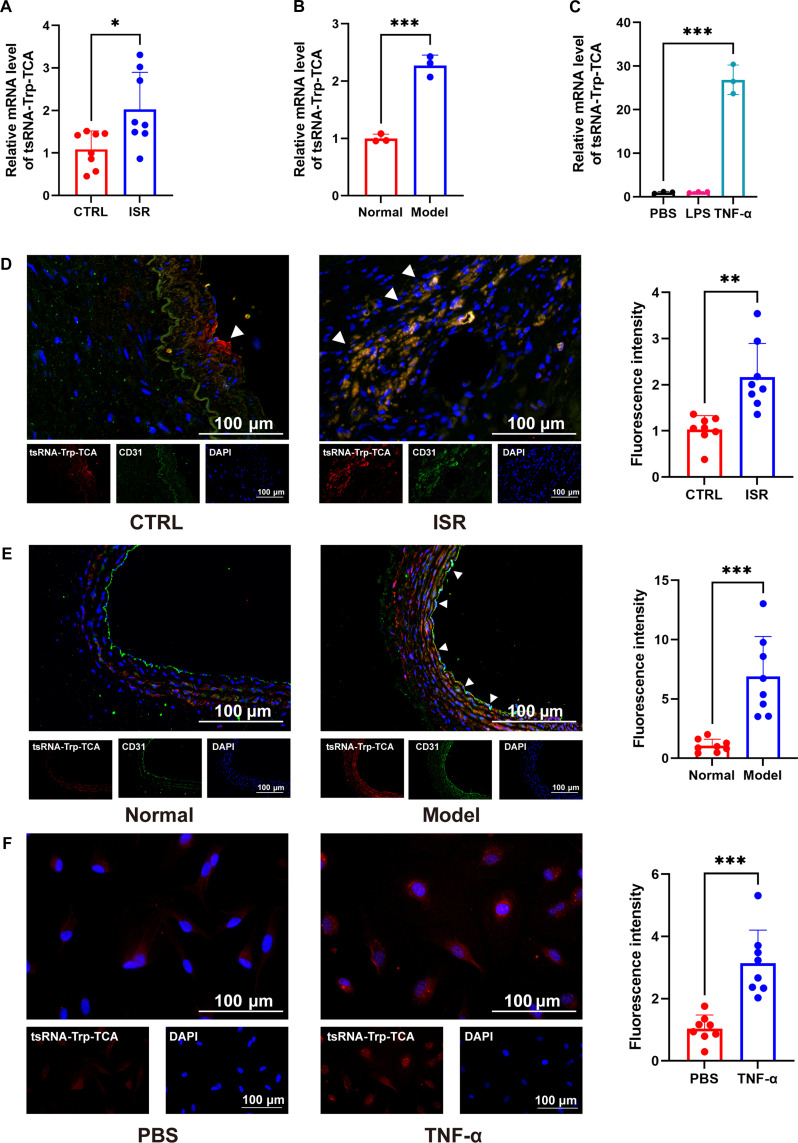
tRF-Trp-TCA is significantly up-regulated in restenosis disease. (A) RT-qPCR for tRF-Trp-TCA in clinical arteries from ISR patients and healthy volunteers (*n* = 8 per group). (B) RT-qPCR for tRF-Trp-TCA in carotid artery balloon injury and normal rat vascular tissues (*n* = 3 per group). (C) RT-qPCR for tRF-Trp-TCA in HUVECs stimulated by LPS and TNF-α. (D to F) FISH immunofluorescence double staining localization of tRF-Trp-TCA in clinical samples (*n* = 3 per group) (D), animal tissues (E), and endothelial cell models (F) (*n* = 8 per group). Data are presented as mean ± SD. *P < 0.05, **P < 0.01, ***P < 0.001.

Based on the tsRNA sequencing data from rat carotid artery balloon injury models, tRF-Trp-TCA exhibited the most marked differential expression between diseased and healthy tissues [26]. Thus, we have identified it as a primary candidate for further investigation. We then validated the differential expression of tRF-Trp-TCA in clinical samples, animal models, and cell models (Fig. 1A to C). The data revealed a significant up-regulation of tRF-Trp-TCA in the diseased tissues of patients with ISR (Fig. 1A). To confirm these findings, we utilized a rat carotid balloon injury model and performed qPCR analysis on the common carotid arteries from both healthy and diseased rats, which corroborated the elevated tRF-Trp-TCA levels in the disease group (Fig. 1B). In both clinical ISR models and animal models of carotid balloon injury, endothelial cell regeneration postinjury is crucial for neointima formation. Therefore, primary human umbilical vein endothelial cells (HUVECs) were stimulated with inflammatory agents such as lipopolysaccharide (LPS) and TNF-α. The results showed that tRF-Trp-TCA expression was most significantly altered under TNF-α stimulation, leading us to focus on TNF-α for further cellular inflammation studies (Fig. 1C). Moreover, a notable disparity was observed in endothelial cells; the elevated expression of tRF-Trp-TCA in endothelial cells was confirmed through fluorescence in situ hybridization (FISH) immunofluorescence double staining of clinical samples, animal tissues, and cells (Fig. 1D to F).

**Fig. 2.**
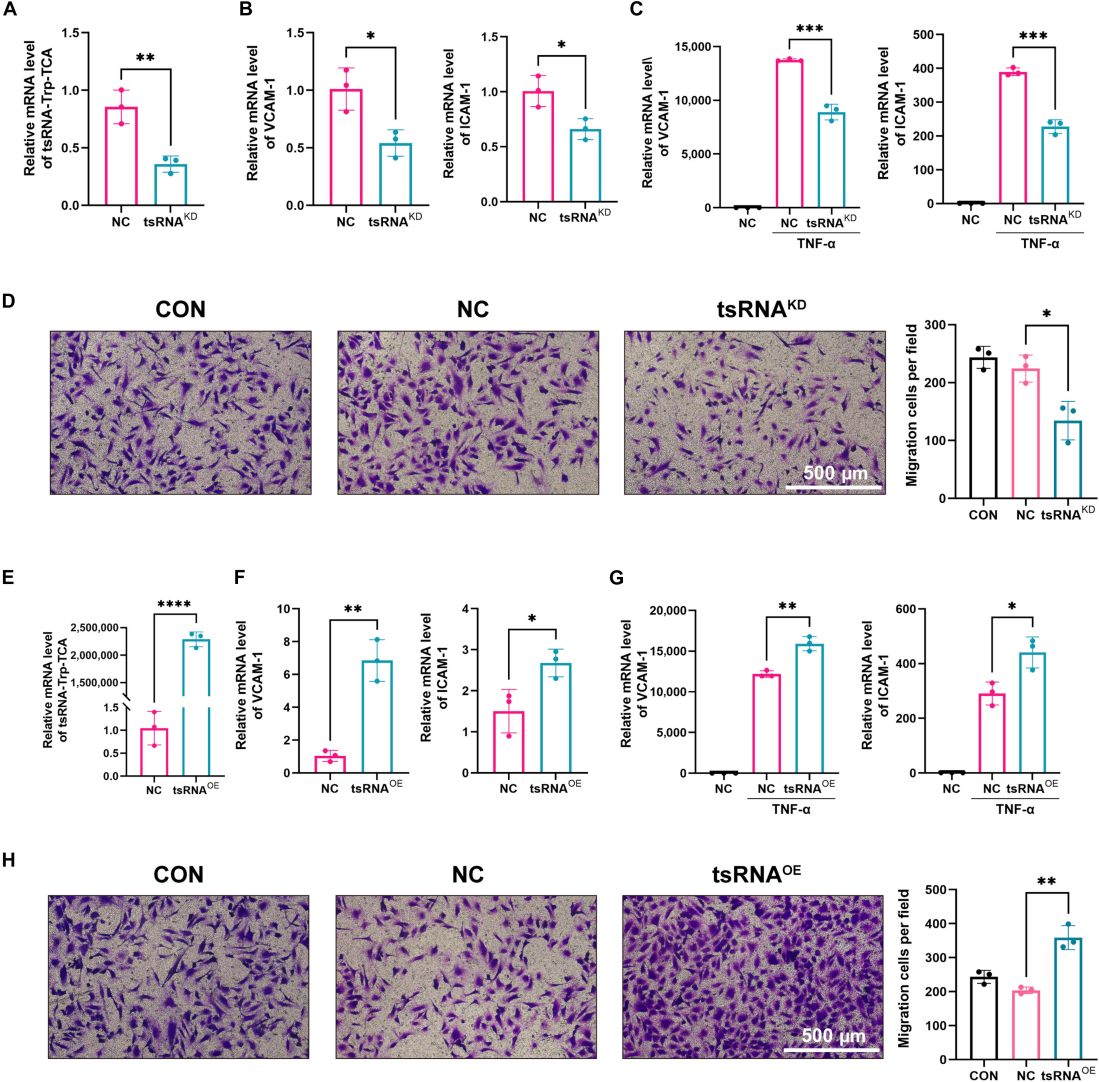
tRF-Trp-TCA is intrinsically linked to the migratory and inflammatory functions of HUVECs. (A) RT-qPCR of tRF-Trp-TCA knockdown in HUVECs. (B) Inflammatory factor expression after tRF-Trp-TCA knockdown under physiological conditions. (C) Inflammatory factor expression after tRF-Trp-TCA knockdown under TNF-α stimulation. (D) Transwell migration assay after tRF-Trp-TCA knockdown in HUVECs. (E) RT-qPCR of tRF-Trp-TCA overexpression in HUVECs. (F) Inflammatory factor expression after overexpression under physiological conditions. (G) Inflammatory factor expression after overexpression under TNF-α stimulation. (H) Transwell migration assay after overexpression in HUVECs. Data are presented as mean ± SD. *n* = 3 per group. *P < 0.05, **P < 0.01, ***P < 0.001.

We subsequently developed a tRF-Trp-TCA inhibitor and effectively silenced tRF-Trp-TCA expression in HUVECs (Fig. 2A). After knockdown of the tRF-Trp-TCA, we examined inflammatory factors ICAM-1 and VCAM-1. The inhibition of tRF-Trp-TCA post-TNF-α stimulation led to a marked reduction in ICAM-1 and VCAM-1 levels, indicating that tRF-Trp-TCA knockdown significantly mitigated TNF-α-induced endothelial cell inflammation (Fig. 2B and C). Recognizing the critical role of endothelial cell proliferation and migration in restenosis progression, we investigated and discovered that tRF-Trp-TCA can modulate the migration and inflammation of HUVECs. Results showed that silencing tRF-Trp-TCA significantly impaired HUVEC migration, as demonstrated by the reduced number of cells in the lower chamber (Fig. 2D). To evaluate the impact of tRF-Trp-TCA on HUVEC proliferation and apoptosis, cellular growth kinetics were examined through Cell Counting Kit-8 (CCK-8) viability assays and 5-ethynyl-2'-deoxyuridine (EdU) incorporation analysis following tsRNA depletion (Fig. S1A and B). Apoptosis markers were analyzed via Western blotting (Fig. S1C). Findings revealed that tRF-Trp-TCA had no significant impact on HUVEC proliferation or apoptosis. Compared with knockdown, the overexpression results showed the opposite trend; tRF-Trp-TCA enhanced HUVEC migratory capacity while up-regulating ICAM-1 and VCAM-1 expression, without exerting significant effects on cellular proliferation or apoptotic pathways (Fig. 2E to H and Fig. S2).

### MEOX2 is a downstream target of tRF-Trp-TCA

**Fig. 3.**
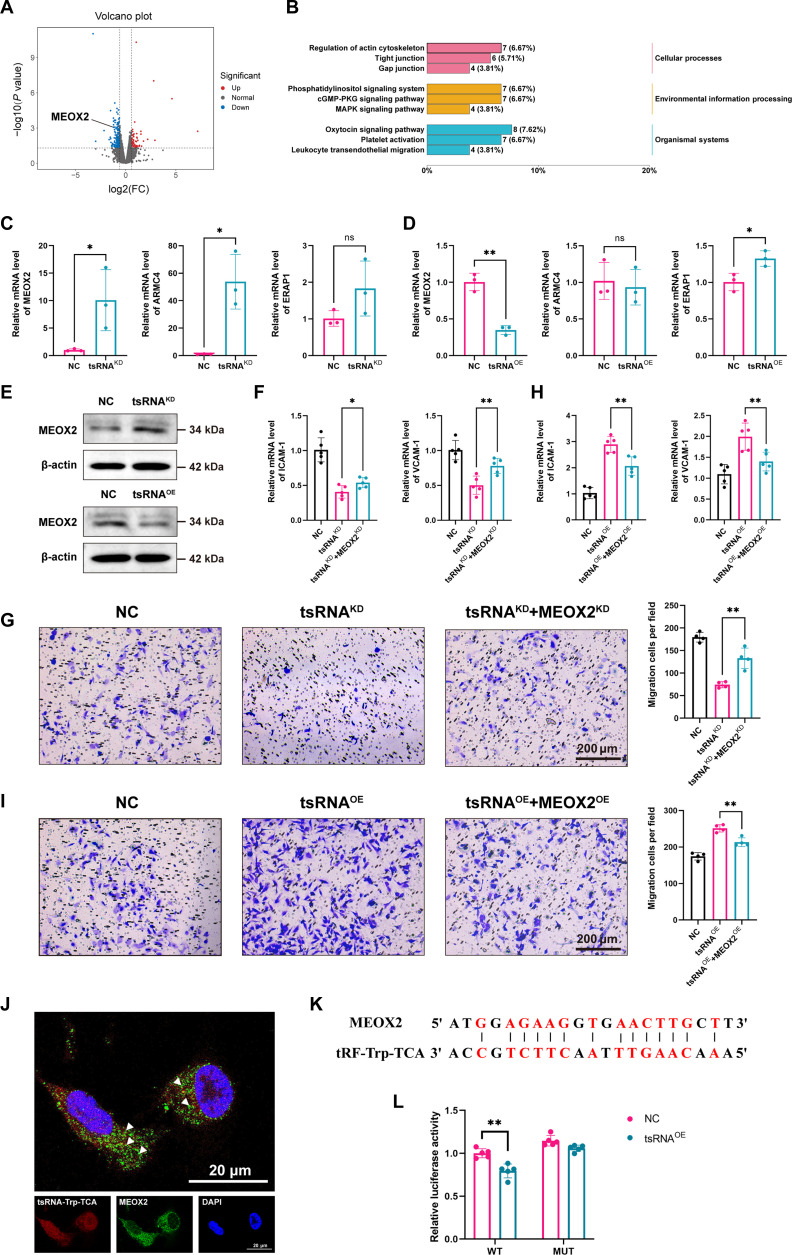
MEOX2 is the genetic downstream target of tRF-Trp-TCA. (A) Volcano plot and (B) key KEGG pathways by RNA sequencing in HUVECs transfected with NC and tRF-Trp-TCA mimics. (C and D) RT-qPCR for RNA expression of MEOX2, ARMC4, and ERAP1MEOX2 after tRF-Trp-TCA knockdown (C) and overexpression (D). (E) Western blot for protein expression of MEOX2 after tRF-Trp-TCA knockdown and overexpression. (F) Inflammatory factor expression after tRF-Trp-TCA knockdown and tRF-Trp-TCA&MEOX2 double-knockdown. (G) Transwell migration assay after tRF-Trp-TCA knockdown and tRF-Trp-TCA&MEOX2 double-knockdown. (H) Inflammatory factor expression after tRF-Trp-TCA overexpression and tRF-Trp-TCA&MEOX2 double-overexpression. (I) Transwell migration assay after tRF-Trp-TCA overexpression and tRF-Trp-TCA&MEOX2 double-overexpression. (J) FISH immunofluorescence double staining localization of tRF-Trp-TCA and MEOX2. (K) The binding site between tRF-Trp-TCA and MEOX2 were predicted theoretically. (L) Dual Luciferase Reporter Gene Assay. Data are presented as mean ± SD. *n* = 3 per group. *P < 0.05, **P < 0.01; ns, no significance.

To clarify the molecular mechanisms underlying the regulatory functions of tRF-Trp-TCA, we conducted RNA sequencing to analyze the differentially expressed genes in HUVECs transfected with negative control (NC) and tRF-Trp-TCA mimic. Among the top 20 down-regulated genes, we analyzed the genes mesenchyme homeobox 2 (MEOX2), ARMC4, and ERAP1, which are linked to cell proliferation, migration, and inflammatory factor expression. Following RT-qPCR validation, only MEOX2 exhibited a trend consistent with the sequencing data. Consequently, we identified MEOX2 as a potential downstream target of tRF-Trp-TCA.MEOX2 (Fig. 3A to D) [45]. The Western blot experiment also demonstrated the differences in the protein level of MEOX2 (Fig. 3E). We subsequently performed a knockout of tRF-Trp-TCA, along with a double knockout of tRF-Trp-TCA and MEOX2, to assess the functional interaction between them. The findings indicated that the knockout of MEOX2 inhibited the effect of tRF-Trp-TCA knockout (Fig. 3F and G). Further experiments involving the overexpression of tRF-Trp-TCA, including dual overexpression assays, reinforced the evidence of their interaction (Fig. 3H and I). Fluorescence colocalization showed their colocalization within the cytoplasm, and then dual luciferase reporter gene assay further confirmed the direct interaction between tRF-Trp-TCA and MEOX2, establishing MEOX2 as the direct target of tRF-Trp-TCA (Fig. 3J to L).

### The acquisition and identification of CELNs

Cabbage, with its high antioxidant content and substantial supply of sap, offers a substantial advantage for the efficient extraction of PELNs in large quantities. Prior research has demonstrated that PELNs derived from cabbage can effectively encapsulate both nucleic acid and chemical drugs, exhibiting notable anti-inflammatory properties [44]. To evaluate the anti-inflammatory properties of CELN, RAW264.7 macrophages were stimulated with LPS and subsequently treated with varying concentrations of CELNs (Fig. S3). The findings demonstrated a marked reduction in the levels of inflammatory cytokines interleukin-1β (IL-1β), IL-6, and TNF-α proportional to the increasing concentration of CELNs. This suggests that CELNs exert a potential inhibitory effect on the inflammatory response. Consequently, we selected cabbage as the source for extracting PELNs.

**Fig. 4.**
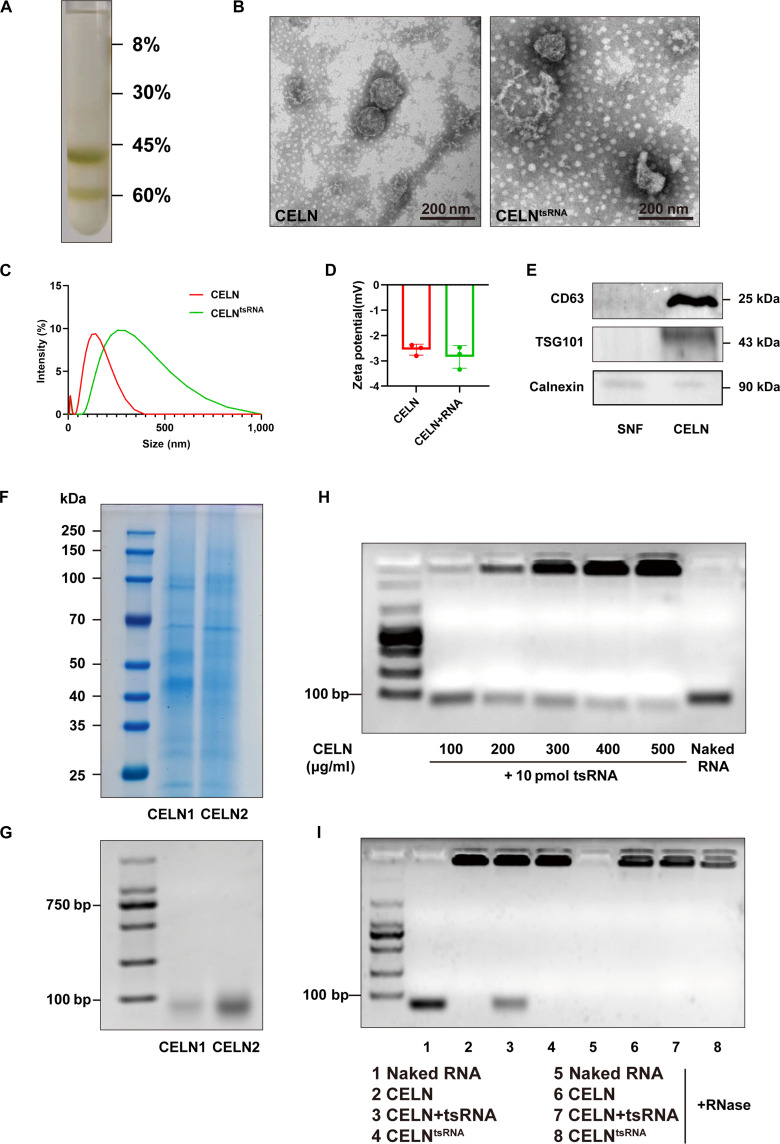
CELN extraction and characterization identification. (A) Stratified plot of sucrose gradient purified CELN. (B) Transmission electron microscopy images of CELN and CELN^tsRNA^. (C) Particle size distribution of CELN and CELN^tsRNA^. (D) Zeta potential analysis of CELN and CELN^tsRNA^. (E) Western blot for CELN marker proteins. (F) Protein Coomassie brilliant blue staining of CELNs. (G) RNA agarose gel electrophoresis of CELNs. (H) CELN loading rate determination. (I) CELN-loaded tsRNA stability assay.

CELN was initially isolated from cabbage juice extracts using differential centrifugation and ultracentrifugation. The purified CELN was obtained through ultracentrifugation with a density gradient sucrose solution (Fig. 4A). Transmission electron microscopy revealed that CELN had a circular shape with a bilayer structure, averaging 40 to 150 nm in diameter (Fig. 4B). Zeta potential analysis showed CELN values between −4 and −2 mV, both pre- and post-drug loading (Fig. 4C and D). These findings confirm the successful isolation of CELNs. Exosomal marker proteins, such as the tetraspanin CD63, tumor susceptibility gene 101 (Tsg101), and negative indicator Calnexin, were identified. Western blotting displayed distinct bands for precipitation-isolated CELNs compared to the supernatant fluid post-ultracentrifugation (Fig. 4E). Coomassie brilliant blue staining and agarose gel electrophoresis of CELN-derived proteins and RNA revealed essential protein and nucleic acid components (Fig. 4F and G). Using a cell electroporator, CELN membranes were disrupted to encapsulate small nucleic acids. The loading efficiency was assessed at various ratios of CELN to nucleic acids, showing nearly complete encapsulation of 10 pmol nucleic acids by 500 μg CELN, with the nucleic acids remaining at the gel top during electrophoresis (Fig. 4H). Post-digestion of free CELN, nucleic acid mixture, and loaded CELN, RNase degraded free nucleic acids while CELN protected the nucleic acids from degradation, maintaining stability (Fig. 4I). To evaluate the release efficiency of nucleic acid drugs encapsulated in CELN, we assessed the release rates at various pH levels. The results showed that in the acidic conditions simulating a lysosome, CELN exhibited a considerably higher release efficiency compared to neutral pH environments, such as simulated blood and cytoplasm. This suggests that CELN effectively maintains the stability of nucleic acids in blood and cytoplasm until it disintegrates upon entering the cytoplasmic lysosome (Fig. S4).

### CELN^tsRNA^ enrichment into the injured carotid artery

**Fig. 5.**
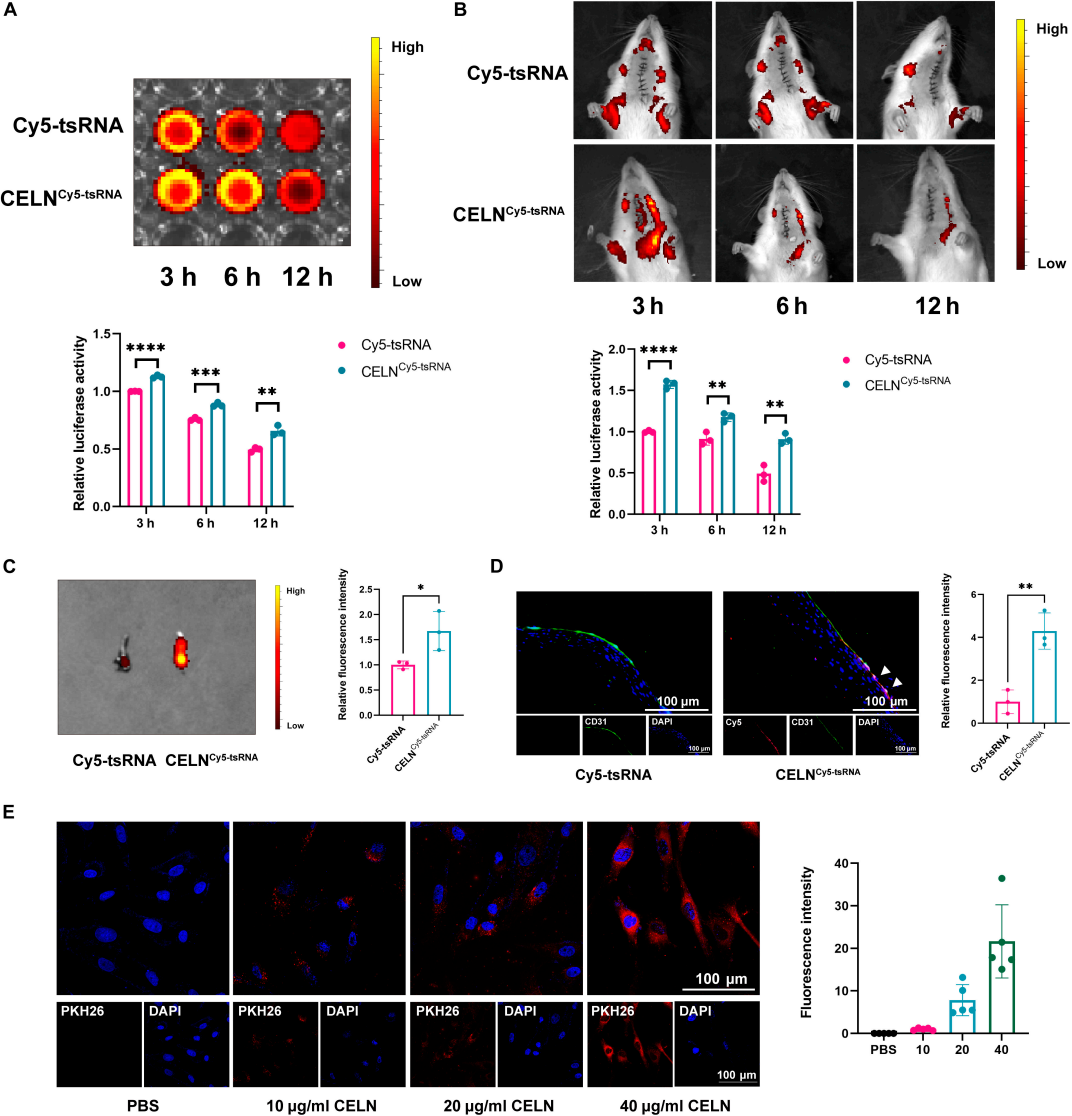
CELN^tsRNA^ is enriched in damaged intima and protects the stability of nucleic acid drugs. (A to C) Blood fluorescence intensity (A), in vivo fluorescence localization (B), and fluorescence intensity (C) of carotid artery detected after injection of CELN and CELN^tsRNA^ in a rat model of carotid artery balloon injury. (D) CD31 immunofluorescent double staining of carotid artery after injury. (E) Fluorescence pattern of cell uptake after incubation of HUVECs with different concentrations of PKH26-labeled CELN. *P < 0.05, **P < 0.01, ***P < 0.001, ****P < 0.0001.

To evaluate the in vivo targeting efficacy of CELN^tsRNA^, we synthesized a Cy5-labeled tsRNA nucleic acid drug and administered it via tail vein injection to rats with carotid balloon injury. Drug metabolism in the bloodstream was monitored using a small animal in vivo imager at 3, 6, and 12 h postinjection. Results showed that CELN significantly inhibited the degradation of nucleic acid drugs in vivo (Fig. 5A). In vivo fluorescence imaging revealed that CELN protection markedly slowed the decline in fluorescence intensity, with notable accumulation at the site of vascular injury in the neck (Fig. 5B). Additionally, the fluorescence intensity within the injured carotid artery tissue was significantly higher in rats treated with the CELN-loaded nucleic acid drug compared to those treated with the free tsRNA nucleic acid drug (Fig. 5C). The distribution of fluorescence in major organs further corroborated the enhanced stability of the CELN-coated tsRNA (Fig. S5). After that, we conducted frozen sections of vascular tissues and concurrently performed CD31 immunofluorescent double staining (Fig. 5D and Fig. S6). The observations revealed that when CELN was encapsulated within tsRNA, endothelial fluorescence was significantly enhanced, suggesting that CELN effectively facilitates the endothelium’s uptake of tsRNA. We further explored the internalization of CELNs by HUVECs since CELNs must bind to endothelial cells to exert their biological effects. HUVECs were cocultured with a medium containing 1 mg of CELNs (Fig. 5E). Fluorescence microscopy images showed that after PKH26 labeling, CELNs were visible in the cytoplasm, and the fluorescence intensity of CELNs uptake increased with higher concentrations. This demonstrates that CELNs can be internalized by vascular endothelial cells. Collectively, our in vivo and in vitro findings demonstrate that CELNs RNA markedly improves targeted delivery to sites of vascular endothelial injury.

### CELN^tsRNA^ significantly alleviated restenosis after carotid artery injury

**Fig. 6.**
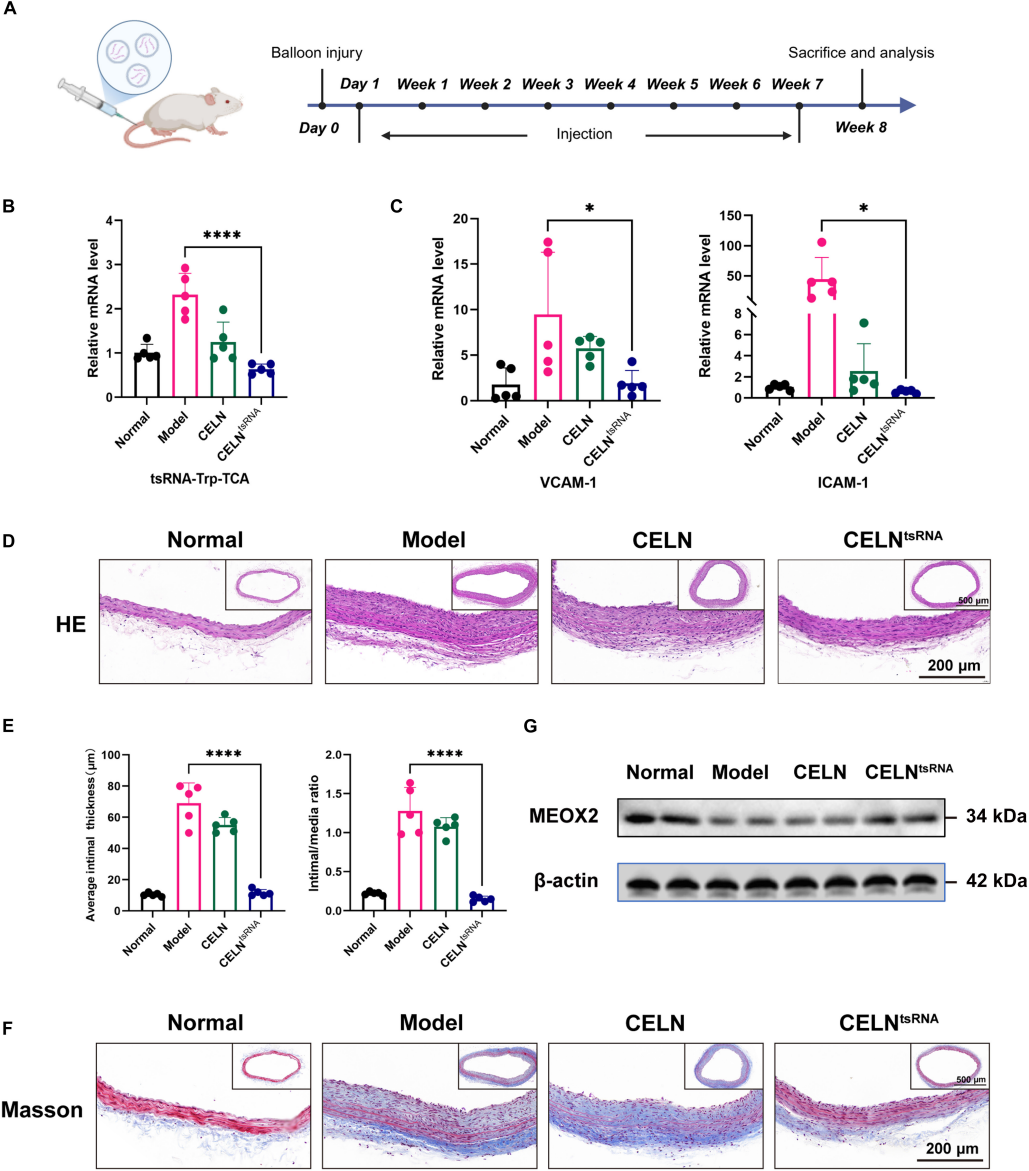
CELN^tsRNA^ significantly inhibited neointimal formation and inflammatory response. (A) Schematic timeline of the animal experiment for in vivo analysis. (B) RT-qPCR for tRF-Trp-TCA in rat carotid artery tissues. (C) RT-qPCR for ICAM-1 and VCAM-1 expression in rat carotid artery tissues. (D) HE staining. (E) Mean intima-media thickness and intima-media ratio. (F) Masson staining. (G) Western blot for MEOX2 expression in rat carotid artery tissues. Data are presented as mean ± SD. *n* = 5 per group. *P < 0.05, ****P < 0.0001.

To evaluate the therapeutic efficacy of CELN loaded with nucleic acid drugs on vascular injury, we developed rat carotid balloon injury models and administered either CELN alone or CELN^tsRNA^ (Fig. 6A). The expression of tRF-Trp-TCA was markedly reduced in the injured carotid arteries of rats treated with CELN^tsRNA^ (Fig. 6B). Analysis of TNF-α downstream genes, ICAM-1 and VCAM-1, revealed a significant down-regulation, indicating that inflammation at the injury site was notably suppressed following CELN^tsRNA^ treatment (Fig. 6C). Hematoxylin and eosin (HE) staining showed a substantial improvement in vascular lumen stenosis, with a marked reduction in neointima thickness compared to the model group (Fig. 6D and E). Furthermore, Masson staining indicated a significant decrease in collagen fiber proliferation posttreatment (Fig. 6F). Following this, we performed a protein-level analysis of the previously validated downstream gene MEOX2. The findings demonstrated that in vivo treatment effectively up-regulated MEOX2 expression, thus mitigating the progression of restenosis (Fig. 6G). In summary, intravenous administration of CELN^tsRNA^ demonstrates a promising therapeutic effect on restenosis following vascular injury.

### CELNs exhibits good biocompatibility

**Fig. 7.**
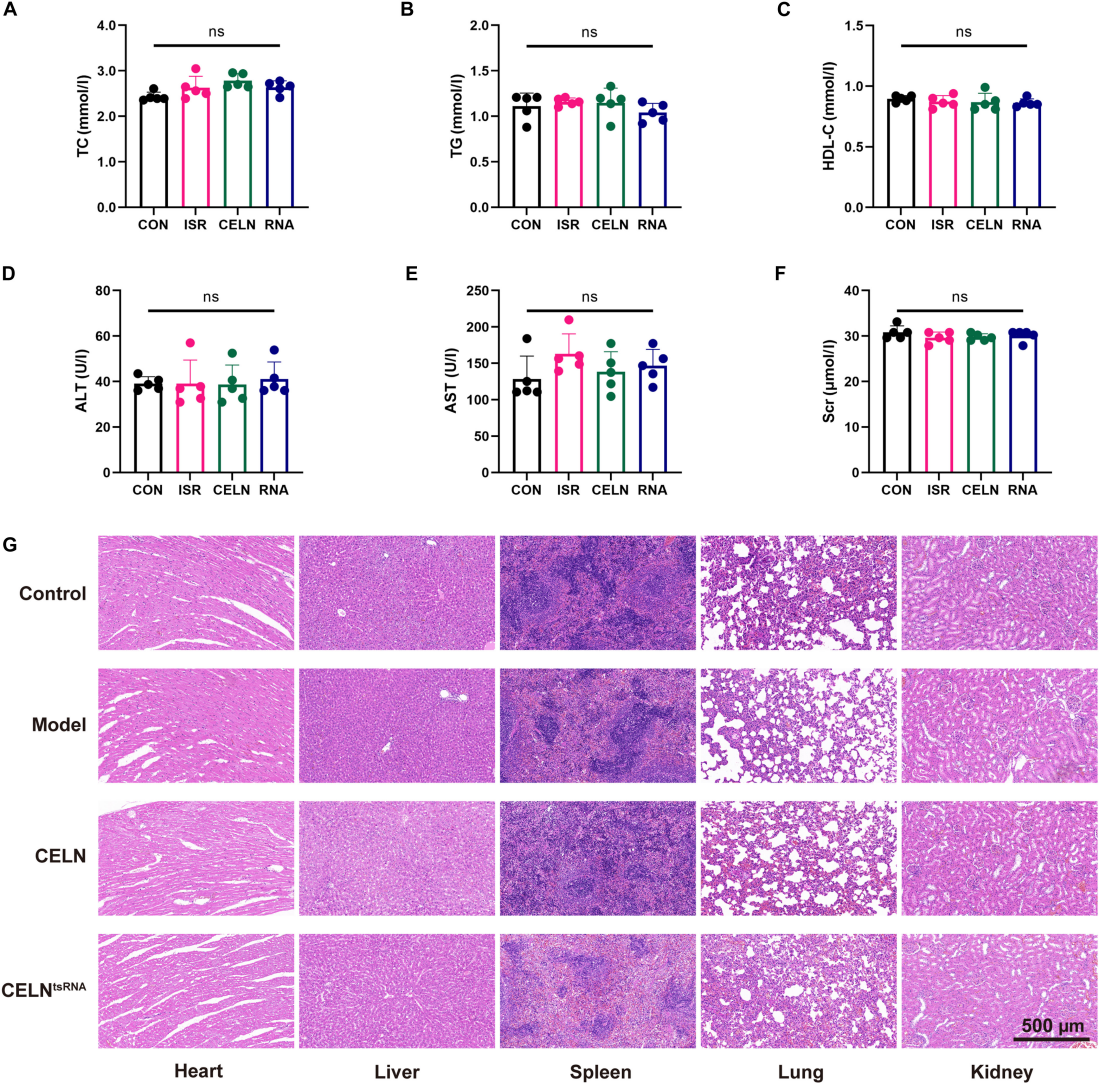
CELN^tsRNA^ exhibits excellent biocompatibility. (A to F) Serum levels of TC (A), TG (B), HDL-C (C), ALT (D), AST (E), and Scr (F). (G) HE staining of sections of heart, liver, spleen, lung, and kidney. Data are presented as mean ± SD. *n* = 5 per group; ns, no significance.

To assess the safety of CELN^tsRNA^, venous blood was collected from the experimental rats after 8 weeks of treatment, and the serum was separated by centrifugation. Serum levels of triglycerides (TG), cholesterol (TC), high-density lipoprotein (HDL-C), alanine aminotransferase (ALT), aspartate aminotransferase (AST), serum creatinine (Scr), and other indicators of hepatic and renal function were analyzed (Fig. 7A to F). The findings demonstrated that neither CELN nor CELN^tsRNA^ significantly impacted these parameters. Following euthanasia, key organs (including cardiac, hepatic, splenic, pulmonary, and renal tissues) were excised and processed for gross morphological evaluation after 8 weeks of treatment (Fig. 7G). Histological analysis using HE staining showed no notable morphological alterations in these organs across the groups, suggesting an absence of significant toxicity to the heart, liver, spleen, lungs, and kidneys. In summary, these data confirm that intravenous administration of CELN^tsRNA^ is not detrimental in vivo.

## Discussion

As a critical technique for treating vascular diseases, endovascular stent implantation is widely utilized. However, restenosis following stent placement remains a marked clinical challenge, severely limiting the therapeutic efficacy for vascular stenosis. Despite the adoption of advanced technologies like drug-eluting stents and radioactive stents, achieving a complete cure for restenosis post-stent implantation remains a pressing issue in medical research. tsRNA, a subclass of noncoding RNA derived from tRNA molecules, holds considerable significance in cardiovascular molecular biology due to its potential impact on CVDs. Recent studies have revealed the complex biological functions of tsRNAs, particularly in relation to vascular diseases [26,27]. In this study, we analyzed transcriptome data from a rat carotid balloon injury model available in public databases. Through differential expression analysis, we identified tRF-Trp-TCA as the most important tsRNA, designating it as a candidate molecule for further investigation. Subsequent research indicates that tRF-Trp-TCA is markedly overexpressed in restenosis models, particularly within the endothelium (Fig. 1). tRF-Trp-TCA plays a critical role in the pathological mechanisms of restenosis by enhancing endothelial cell migration and up-regulating inflammatory factor expression (Fig. 2).

MEOX2inhibits inflammation and fibrosis in vascular endothelial cells as a transcription factor [46]. MEOX2 not only attenuates the angiogenic response of ECs to proangiogenic factors but also inhibits their activation by chronic inflammatory stimuli that typically engage NF-κB pathways [47]. We confirmed the interaction and functional relationship between the two, demonstrating that MEOX2 is a direct downstream regulatory target of tRF-Trp-TCA (Fig. 3).

PELNs comprise various bioactive and therapeutic agents. Recent research has underscored their potential as effective modalities for disease prevention and intervention. Plants such as ginger, green tea, and pomegranate are utilized for their various therapeutic properties, leading to the incorporation of their PELNs for the delivery of specific miRNAs or pharmaceutical compounds to achieve therapeutic outcomes [39,40,48,49]. Prior studies revealed that CELNs possess the ability to modulate inflammation in immune cells while also preventing apoptosis in human keratinocytes and fibroblasts [44]. Simultaneously, the benefits of its botanical extract, including high yield, cost-effectiveness, and consistent release, offer specific advantages in yield, stability, and bioactivity, supporting its advancement toward practical applications. Furthermore, pharmaceutical agents encapsulated within these nanovesicles were effectively internalized by target human cells, with subsequent release and therapeutic activity, confirming their utility as biocompatible drug delivery vehicles. This study effectively isolated CELNs from cabbage and assessed their properties and potential for nucleic acid drug delivery (Fig. 4). Intravenous administration of fluorescently tagged CELN^tsRNA^ revealed their selective accumulation in damaged arterial tissues (Fig. 5). Therapeutic evaluations demonstrated that CELN^tsRNA^ can reduce arterial inflammation and neointimal hyperplasia, while also exhibiting favorable biocompatibility (Figs. 6 and 7).

Our experimental results validate the significant targeting and therapeutic potential of CELN^tsRNA^; however, several critical issues require urgent resolution. Firstly, the active targeting mechanism of CELN in restenosis remains undefined. While PELN, as a natural nanoparticle, exhibits passive targeting capabilities, its abundant surface components might confer active targeting properties, which are currently under rigorous investigation. Secondly, the therapeutic functions of CELN are not fully elucidated. Although previous studies indicate CELN’s anti-inflammatory and antioxidant properties, the mechanisms underlying its interactions with nucleic acid drugs are not well understood, suggesting a potential synergistic effect. Additionally, there is still potential to enhance the loading rate, stability, and delivery efficiency of CELNs, particularly by incorporating functional groups into the CELNs lipid membrane. The analysis does not adequately cover critical aspects such as pharmacokinetics and immunology. Specific immune-related adverse events, including complement activation, cytokine release, and long-term immunogenicity, remain unassessed. Long-term drug toxicity and large animal experiments are also lacking in supplementation. These deficiencies necessitate additional experimental investigation and research.

## Conclusion

In summary, we investigated the pathogenesis of tRF-Trp-TCA in post-vascular injury restenosis and targeted it for intimal hyperplasia and inflammation therapy. We developed an innovative antagomiR-tRF-Trp-TCA delivery system utilizing cabbage-derived PELN. This system enables effective nucleic acid drug delivery for treating vascular restenosis and may foster advancements in novel bioderived vesicular nucleic acid therapeutics.

## Materials and Methods

### Participants sample collection

This study was conducted in full adherence to the ethical standards established by the Declaration of Helsinki and received formal approval from the Institutional Review Board of Qingdao University (QYFYWZLL29942). Participants provided informed consent after being fully briefed on the research aims. Between September 2022 and September 2023, the research team recruited 8 patients who developed restenosis following femoral artery stenting at the Affiliated Hospital of Qingdao University. These patients were matched with 8 healthy volunteers as controls. Restenotic vessels were excised post-amputation, while normal vessels were obtained from organ donors. Each patient with ISR underwent lower extremity arteriography and femoral artery stenting. The relevant patient information is listed in Table S1.

### RNA isolation

For each tissue sample, total RNA extraction was performed with TRIzol Reagent (Life Technologies, California, USA) following the supplier’s protocol. RNA concentration and purity were determined using a NanoDrop 2000 spectrophotometer (Thermo Fisher Scientific, Wilmington, DE). To ensure RNA stability and maintain clinical utility, specimens were stored in RNase-free tubes at −80 °C prior to downstream assays.

### Rat carotid artery balloon injury model

Animal experiments were conducted under the approval of the Institutional Animal Ethics Committee of Qingdao University (20240610SD2420240901178) and in line with the NIH Guide for the Care and Use of Laboratory Animals. Rat carotid artery injury was produced using established methods. Male Sprague–Dawley rats aged 8 weeks, sourced from Beijing HFK Bioscience Co., Ltd. (Beijing, China), received pentobarbital sodium anesthesia (50 mg/kg, i.p.). Through a midline neck incision, the left common carotid artery and its proximal portion were exposed. The external carotid artery was then ligated and transected, and a 2F Fogarty embolectomy catheter was advanced retrogradely. The Fogarty catheter balloon was inflated to 1.5 atmospheric pressure and gently withdrawn with oscillating motion to induce controlled endothelial denudation. Following injury induction, the external carotid artery stump was permanently ligated to re-establish antegrade flow through the common carotid artery.

### Cell culture, transfection, and treatment

HUVECs were obtained from the American Type Culture Collection (ATCC) and cultured in Gibco’s Medium 199 (M199) supplemented with 15% fetal bovine serum (FBS) from Inner Mongolia Opcel Biotechnology Co., Ltd., China, and 1% penicillin/streptomycin from Meilun, Dalian, China. The cells were incubated in a humidified environment at 37 °C with 5% CO_2_. Transfection was conducted using Lipofectamine 3000 (Invitrogen, Carlsbad, CA, USA) according to the manufacturer’s protocol. The sequences of the mimics and inhibitors targeting tRF-Trp-TCA (GenePharma, Shanghai, China) are detailed in Table S2. For TNF-α induction, HUVECs were exposed to 200 ng/ml in basal medium (MCE, NJ, USA) for 24 h.

### Fluorescence in situ hybridization

FISH was performed as previously reported [50]. The Cy5-labeled specific tRF-Trp-TCA probe, produced by GenePharma (Shanghai, China), was employed alongside the GenePharma FISH kit. Following the manufacturer’s instructions, samples were mounted on slides and fixed in 4% paraformaldehyde at 25 °C for 20 min. Subsequently, they were treated with protease K at 37 °C for 20 min, then incubated in a denaturation solution at 78 °C for 8 min. Hybridization with the fluorescent probe was carried out at 37 °C for 12 h, followed by a 15-min wash. Nuclei were stained with 4',6-diamidino-2-phenylindole (DAPI), and imaging was conducted using a Nikon confocal microscope. The probe sequences are provided in Table S3.

### RNA sequencing

HUVECs were transfected with NC and tRF-Trp-TCA mimic, followed by RNA extraction for RNA sequencing library preparation, as previously outlined. Libraries were constructed using the RNA Library Prep Kit (NEB, USA) in accordance with the manufacturer’s protocol. Subsequently, library samples underwent clustering with the TruSeq PE Cluster Kit v3-cBot-HS (Illumina, USA) and were paired-end sequenced on an Illumina NovaSeq 6000 system equipped with S4 flow cells.

### Dual luciferase reporter gene assay

A segment of either the Western ant (Mut) or wild-type (Wt) sequences, both containing the tRF-Trp-TCA and MEOX2 binding sites, was inserted into the pmirGLO vector (Genechem, Shanghai, China) at the SalI and SacI restriction sites. We then cotransfected the modified luciferase vector with either the tRF-Trp-TCA mimic or NC using Lipofectamine 3000. Subsequently, luciferase activity was measured with the Firefly & Renilla-Glo Luciferase Reporter Assay Kit (Genechem, Shanghai, China).

### Preparation of CELNs and CELN^tsRNA^

CELNs were isolated from cabbages obtained from a local market. The cabbages were thoroughly washed with distilled water 3 times to remove any contaminants such as dust, dirt, and pesticides. The cabbage juice was then extracted using a juicer and subjected to density gradient centrifugation at 300×*g*, 3,000×*g*, and 10,000×*g* for 30 min each to eliminate grinding and cellular debris. CELNs were subsequently isolated by ultracentrifugation (Optima XPN-100, Beckman, USA) at 150,000×*g* for 1 h. Following centrifugation, the supernatant was carefully removed and discarded, while the pellet containing vesicles was gently resuspended in sterile 1× phosphate-buffered saline (PBS, pH 7.4). A series of sucrose solutions (8%, 30%, 45%, and 60%) in 20 mM Tris-HCl were layered in an ultracentrifuge tube according to their density gradient. The CELN suspension was added and centrifuged again at 150,000×*g* for 1 h for further purification. The fractions from the 45% and 60% sucrose layers were collected, re-suspended, and yielded purified CELNs. The protein concentration of the CELNs was quantified using the bicinchoninic acid (BCA) assay.

As described in the previous article, electroporation utilizes an electric field to induce transient hydrophilic pores in PELN membranes via pulsed current, enabling tsRNA to enter CELNs. The CELNs were isolated by ultracentrifugation and stored at −80 °C post-purification. Concurrently, 10 pmol of tsRNA and different concentrations of PELNs were prepared for electroporation using the Gene Pulser Xcell System (Bio-Rad, USA) under conditions of 1,000 V and 50 μF, in an electroporation buffer containing 400 mM sucrose in PBS. The samples were incubated at 37 °C for 1 h and then at 4 °C overnight. A second round of ultracentrifugation was performed to separate the precipitated CELNs and eliminate any unencapsulated nucleic acids.

### Characterization of CELNs and CELN^tsRNA^

Sample morphology and integrity were assessed via transmission electron microscopy. For this purpose, 200-mesh nickel grids coated with formvar carbon were prepared with CELNs and incubated for 20 min. Subsequently, the samples were treated with 1% phosphotungstic acid for staining and were imaged using a Regulus8100 microscope (Hitachi, Japan). For zeta potential and size characterization, CELNs were analyzed with a Zetasizer Nano ZS (Malvern, UK), with measurements conducted in triplicate to ensure precision.

### Coomassie brilliant blue staining and agarose gel electrophoresis

Protein was extracted from CELNs using RIPA lysis buffer (Solarbio, Beijing, China) and subsequently analyzed via sodium dodecyl sulfate–polyacrylamide gel electrophoresis (SDS-PAGE), followed by Coomassie brilliant blue staining (Solarbio, Beijing, China). For the evaluation of CELNs and CELNtsRNA, a 2% agarose gel containing ethidium bromide was utilized. The samples underwent electrophoresis in Tris-acetate-EDTA (TAE) buffer (Solarbio, Beijing, China) at 120 V for 45 min with a mini-sub cell GT (Bio-Rad, USA). Visualization of the gel was accomplished using the Fusion Solo S system (Vilber Lourmat, Germany).

### Release efficiency of CELN^tsRNA^

CELN was solubilized in a buffer comprising acetic acid and sodium acetate at pH 5.0, as well as in a PBS solution at pH 7.4. At various time intervals, CLEN and the solution’s supernatant were isolated, and the tsRNA content in each component was quantified. The release rate was determined by calculating the percentage of tsRNA in the supernatant relative to the total tsRNA present.

### Real-time quantitative PCR

Real-time quantitative polymerase chain reaction (RT-qPCR) was performed as previously reported [51]. After extracting and quantifying RNA, complementary DNA (cDNA) was synthesized using the cDNA Synthesis SuperMix reverse transcription kit from Shanghai Yisheng Biotechnology Co., Ltd. The synthesis of the first strand of miRNA cDNA for tRF-Trp-TCA inversion was conducted with the stem-loop method according to the miRNA cDNA first-strand synthesis kit instructions from Hunan Ecoray Bioengineering Co., Ltd. Relative expression levels of target genes were quantified using RT-qPCR, employing U6 or GAPDH as internal controls and SYBR Green Master Mix for the assays. Primer sequences are listed in Table S4. To ensure result accuracy, all protocols were executed in strict accordance with the manufacturer’s guidelines. The comparative 2^−ΔΔCt^ method was applied to calculate relative gene expression levels.

### Western blot

Western blot was performed as previously reported [52]. Proteins isolated from the vascular tissues of both healthy and diseased subjects were analyzed using SDS-PAGE, followed by transfer to a 0.22-μm polyvinylidene fluoride (PVDF) membrane (Millipore, USA). To reduce nonspecific binding of antibodies, the membrane was blocked for 1 h at room temperature (25 °C) with 5% (w/v) nonfat dry milk in Tris-buffered saline containing 0.1% Tween-20 (TBST) and gently agitated. The membrane was then incubated overnight at 4 °C with the following primary antibodies: β-actin (Absin, abs132001, 1:5,000), BAX (PTM Biolab, PTM 5482, 1:1,000), BCL2 (PTM Biolab, PTM 5777, 1:1,000), NLRP3 (Boster, PB0121, 1:2,000), GPX4 (PTM Biolab, PTM 6775, 1:1,000), CD63 (Absin, abs132700, 1:2,000), and Tsg101 (Absin, abs122785, 1:2,000). Subsequently, the membrane was treated with a secondary antibody against mouse IgG (H+L) (Jackson ImmunoResearch, Philadelphia, USA, 1:5,000) for 1 h at room temperature. Finally, protein bands were visualized using the Fusion Solo S imaging system (Vilber Lourmat, Germany).

### CCK-8 assay

The proliferation of HUVECs was assessed using the CCK-8 assay from Shanghai Yisheng Biotechnology Co., Ltd. HUVECs were transfected in 96-well plates for 24 h. After treatment, 10 μl of CCK-8 solution was added to each well with a multichannel pipette, followed by incubation for 2 h at 37 °C in a 5% CO₂ humidified environment. Absorbance at 450 nm, indicative of cell proliferation, was subsequently measured. Each experiment was conducted in quintuplicate.

### Transwell assay

Transwell assay was performed as previously reported [53]. Following transfection, HUVECs were incubated in 6-well plates at 37 °C with 5% CO_2_ for 24 h. Subsequently, the cells were placed in serum-free M199 for 6 h before being transferred to the upper chamber of a transwell system (Corning, NY, USA) containing 200 μl of serum-free medium. The lower chamber was filled with 500 μl of M199 enriched with 15% FBS and allowed to incubate for an additional 24 h. Afterward, cells were fixed using 4% paraformaldehyde for 1 h and stained with 0.1% crystal violet for 30 min. Migrated cells were visualized using a microscope (Nikon, Japan). All experiments were conducted in triplicate.

### Cellular uptake assay

The PKH-26 dye (AbMole, USA) was freshly prepared and subsequently combined with CELNs, followed by a 30-min incubation at 37 °C. To eliminate any unbound dye, ultracentrifugation was conducted for 90 min. The resulting labeled CELNs were then suspended in M199 medium to culture HUVECs for 24 h, after which cellular uptake was assessed using a Nikon confocal laser scanning microscope.

### In vivo targeting capability

Two weeks post-carotid balloon injury, male SD rats were randomly divided into 2 groups: one treated with Cy5-tsRNA and the other with CELNCy5-tsRNA, administered via tail vein injection (0.5 mg/kg). After 24 h, rats were anesthetized with 2% isoflurane, and sample distribution was analyzed using the in vivo imaging system (IVIS) (PerkinElmer, USA). After euthanasia, carotid arteries and major organs—including the heart, liver, spleen, lungs, and kidneys—were excised. Blood samples were drawn from the tail vein and placed into a 96-well plate. The fluorescence intensity of the blood, carotid arteries, and organs was assessed using the IVIS system. Carotid tissues were embedded in optimal cutting temperature (OCT) compound and sectioned into 8-μm slices. These sections were fixed, treated with an antifluorescence quenching sealer containing DAPI, and subsequently imaged with a fluorescence microscope (Nikon, Japan).

### HE staining

Eight weeks after surgery, the vessel tissues were fixed in 4% paraformaldehyde for 24 h, followed by embedding in paraffin (Thermo Fisher Scientific, USA) at 65 °C for 12 h. Subsequently, 8-μm sections were obtained using a paraffin microtome (Leica, Germany) and prepared for HE staining (Solarbio, Beijing, China). The stained tissue images were then acquired using a Nikon microscope (Japan).

### Masson staining

The paraffin sections were initially dewaxed and rehydrated, followed by rinsing 2 to 3 times with both tap and distilled water. The nuclei were stained using Weigert’s hematoxylin for 5 to 10 min, then differentiated with hydrochloric acid alcohol and washed 3 times with distilled water. Next, the sections underwent treatment with Masson Ponceau acid-decolorizing solution for 5 to 10 min and were immersed in a 2% glacial acetic acid solution for 10 min. Differentiation continued with a 1% phosphomolybdate solution for 3 to 5 min without subsequent washing, followed by staining with aniline blue solution for 5 min. The sections were then placed in a 0.2% glacial acetic acid solution for 2 min. Finally, after dehydration and clearing, the sections were mounted with neutral gum. This staining technique highlighted collagen fibers, mucus, and cartilage in blue, while cytoplasm, muscle, cellulose, and glial cells were stained red, and the nuclei exhibited black and blue tones.

### Serum safety assessment of CELNs in vivo

Scr, TC, TG, HDL-C, AST/GOT, and ALT/GPT were quantified using standardized commercial assay kits from Nanjing Jiancheng Bioengineering Institute, China, adhering to the manufacturer’s optimized protocols. Optical density (OD) values were calculated by subtracting the OD of the control wells from those of the sample wells. The resultant corrected OD values were then analyzed against a standard curve to establish specific activity units.

### Statistical analysis

All experimental data were analyzed with GraphPad Prism 9.0. Continuous variables are presented as means ± standard deviations (SD). Statistical analyses included Student’s *t* test and 1-way and 2-way analysis of variance to assess intergroup differences. A *P* value of less than 0.05 was deemed statistically significant, indicated by the following notation: *P ≤ 0.05, ** *P* ≤ 0.01, *** *P* ≤ 0.001.

## Data Availability

The data are freely available upon request.
