# Supplementary material for: Cabbage Exosome-Like Nanoparticles Encapsulating Small Noncoding tsRNA Prevent Postinjury Arterial Restenosis
Source: Research (Wash D C). 2025 Nov 26;8:1019. doi: 10.34133/research.1019 (PMC12648583; doi:10.34133/research.1019)
Supplement: Supplementary 1 — Tables S1 to S4 Figs. S1 to S6 [file research.1019.f1.docx]

**Supplementary Materials**

**Table S1 Patient information**

| **Characteristic** | **ISR Patients (n=8)** | **Healthy Controls (n=8)** | **P Value** |
| --- | --- | --- | --- |
| **Age (years, Mean ± SD)** | 58.9 ± 6.1 | 50.5 ± 7.3 | 0.018 |
| **Gender (Male: Female)** | 6:2 | 4:4 | 0.350 |
| **Hypertension, n (%)** | 6 (75.0%) | 2 (25.0%) | 0.141 |
| **Diabetes, n (%)** | 4 (50.0%) | 1 (12.5%) | 0.282 |
| **Hyperlipidemia, n (%)** | 7 (87.5%) | 2 (25.0%) | 0.026 |
| **Smoking History, n (%)** | 5 (62.5%) | 2 (25.0%) | 0.330 |

**Table S2 tRF-Trp-TCA inhibitor, mimic and siRNA of MEOX2**

| **Name** | **Sequence（5´-3´）** |
| --- | --- |
| tRF-Trp-TCA mimic | Sense: AAA CAA GUU UAA CUU CUG CCA  Antisense: GCA GAA GUU AAA CUU GUU UUU |
| mimic-NC | Sense: UUC UCC GAA CGU GUC ACG UTT  Antisense: ACG UGA CAC GUU CGG AGA ATT |
| tRF-Trp-TCA inhibitor | UGG CAG AAG UUA AAC UUG UUU |
| inhibitor-NC | CAG UAC UUU UGU GUA GUA GUA GUA GUA CAA |
| siRNA-NC | Sense: UUC UCC GAA CGU GUC ACG UTT  Antisense: ACG UGA CAC GUU CGG AGA ATT |
| siRNA-MEOX2 | Sense: GGA CUC UAU AGC AAA UGA ATT  Antisense: UUC AUU UGC UAU AGA GUC CTT |

**Table S3 FISH probe sequence**

| **Probe name** | **Probe sequence（5´-3´）** |
| --- | --- |
| tRF-Trp-TCA | AAA CAA GTT TAA CTT CTG CCA |
| NC | TGC TTT GCA CGG TAA CGC CTG TTT T |

**Table S4 PCR primer sequence**

| **Primer name** | **Primer sequence（5´-3´）** |
| --- | --- |
| tRF-Trp-TCA-rt | GTCGTATCCAGTGCAGGGTCCGAGGTATTCGCACTGGATACGACTGGCAG |
| tRF-Trp-TCA-F | CGCGCGAAACAAGTTTAACTT |
| Universal-R  VCAM-1-F | AGTGCAGGGTCCGAGGTATT  TCTCATTGACTTGCAGCACCACAG |
| VCAM-1-R | CTCCATCGTCACCTTCCCATTCAG |
| ICAM-1-F | GGTAGCAGCCGCAGTCATAATGG |
| ICAM-1-R | GTGGCTTGTGTGTTCGGTTTCATG |
| GAPDH-F | GCACCGTCAAGGCTGAGAAC |
| GAPDH-R | TGGTGAAGACGCCAGTGGA |
| U6-F | GGAACGATACAGAGAAGATTAGC |
| U6-R | TGGAACGCTTCACGAATTTGCG |
| IL-1β -F | ATGATGGCTTATTACAGTGGCAA |
| IL-1β -R | GTCGGAGATTCGTAGCTGGA |
| IL-6-F | ACTCACCTCTTCAGAACGAATTGG |
| IL-6-R | CCATCTTTGGAAGGTTCAGGTTGG |
| ARMC4-F | AATCCTCGAAATCACCCCTCT |
| ARMC4-R | CGCCAAACTTGTGTTCCATTC |
| ERAP1-F | CCCCTCAAATGGTCCCTTGC |
| ERAP1-R | GAGATGCTTCAGTGCTCTGAC |
| MEOX2-F | GCACCCGTTCTCCCAATCC |
| MEOX2-R | TCCCGCGATTATGCAAGATGA |


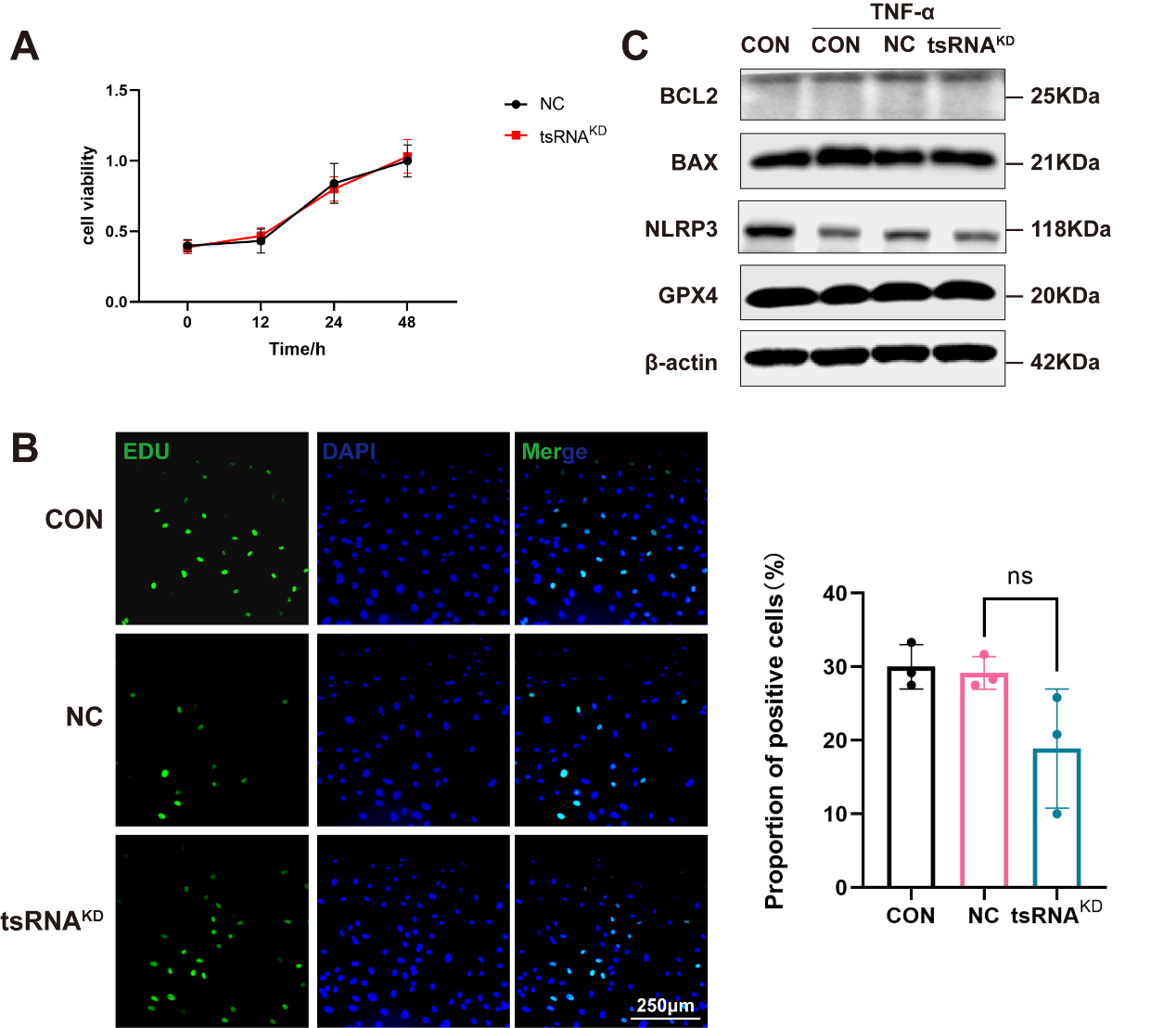
**Figure S1 HUVECs proliferation and apoptosis after tRF-Trp-TCA knockdown**

(A) CCK8 assay after tRF-Trp-TCA knockdown; (B) Apoptosis factor expression after tRF-Trp-TCA knockdown under TNF-α stimulation; (D) EdU assay after tRF-Trp-TCA knockdown in HUVECs. Data are presented as mean ± SD. n=3 per group. ns, no significance.


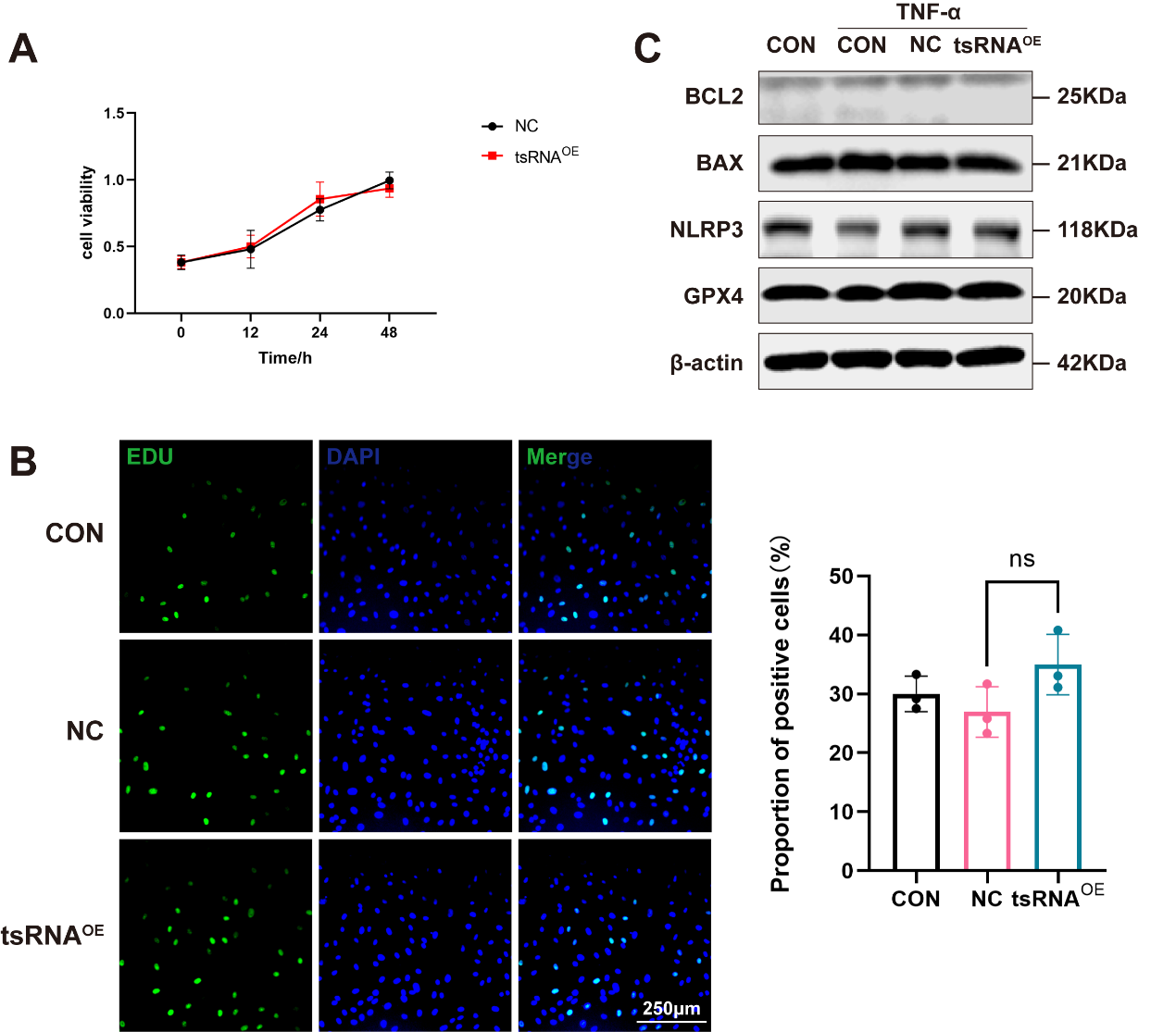
**Figure S2 HUVECs proliferation and apoptosis after tRF-Trp-TCA overexpression**

(A) CCK8 assay after tRF-Trp-TCA overexpression; (B) Apoptosis factor expression after tRF-Trp-TCA overexpression under TNF-α stimulation; (D) EdU assay after tRF-Trp-TCA overexpression in HUVECs. Data are presented as mean ± SD. n=3 per group. ns, no significance.


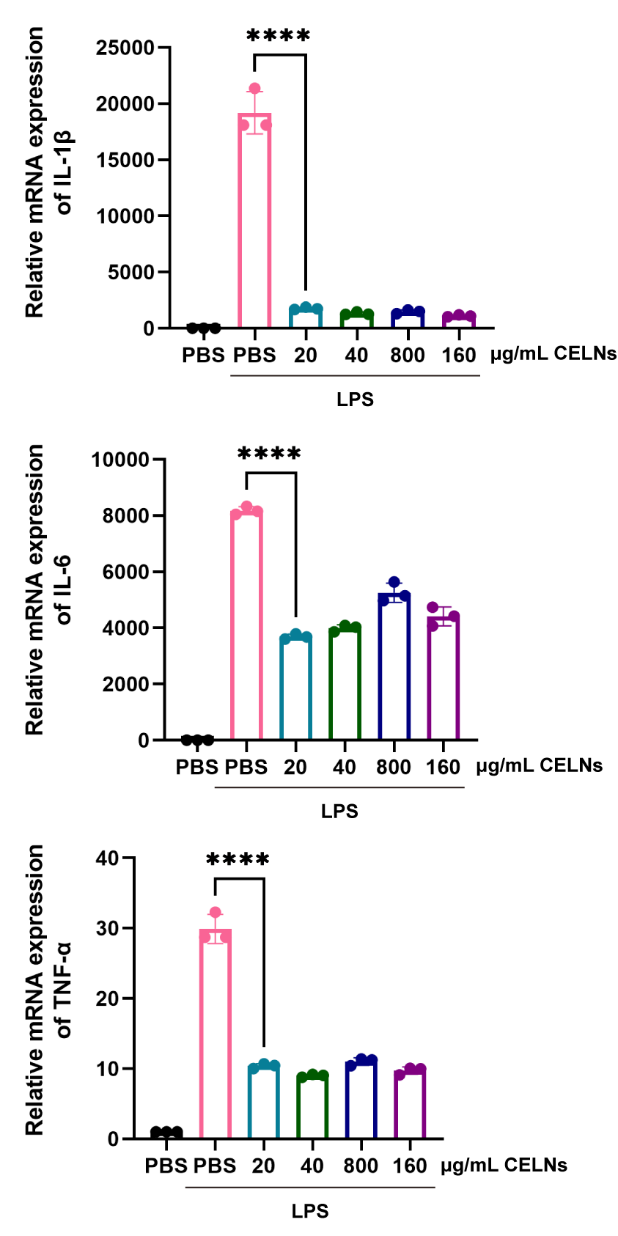


**Figure S3 CELNs has certain anti-inflammatory ability**

RT-qPCR of IL-1β, IL-6 and TNF-α after treated with LPS and CELNs in RAWs.


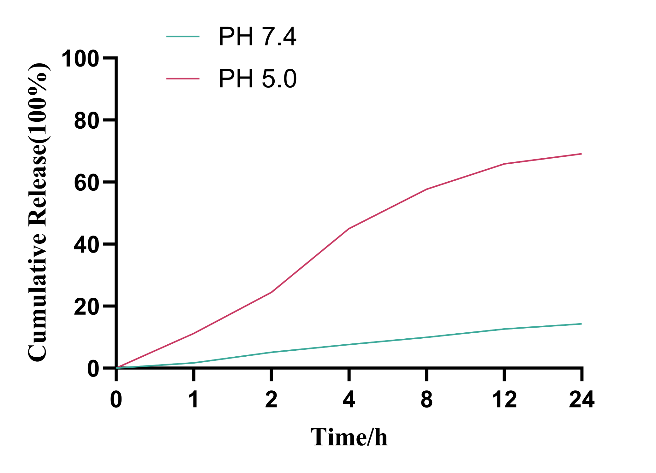


**Figure S4 Release rate of CELN^tsRNA^**

The speed and efficiency of CELN^tsRNA^ release in different environments.


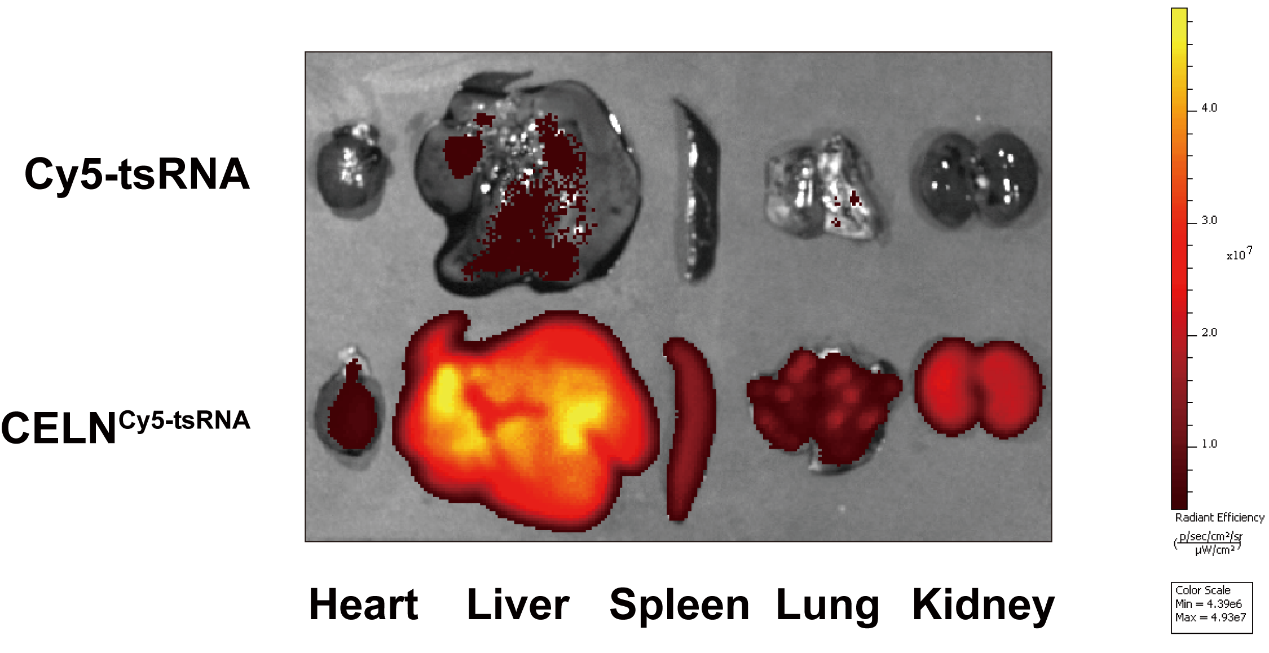


**Figure S5 Main organ fluorescence distribution**

Fluorescence intensity of heart, liver, spleen, lung and kidney detected after injection of CELN and CELN^tsRNA^ in rat model of carotid artery balloon injury.


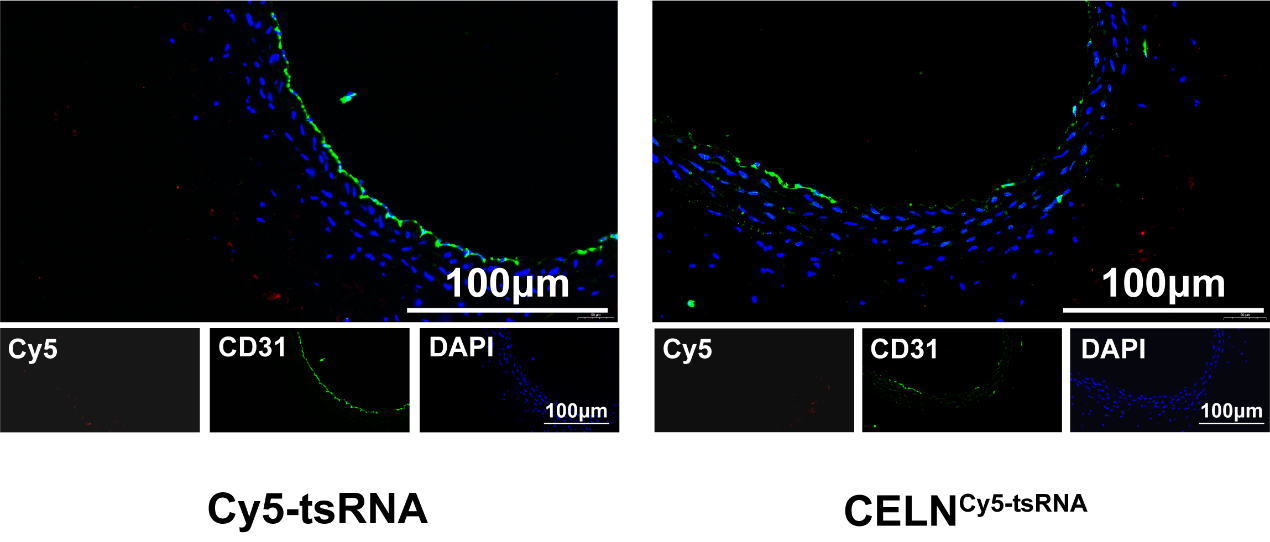


**Figure S6 Uptake of CELN^tsRNA^ in healthy carotid artery**

CD31 immunofluorescent double staining of healthy carotid artery
